# AIQM2: organic reaction simulations beyond DFT

**DOI:** 10.1039/d5sc02802g

**Published:** 2025-08-07

**Authors:** Yuxinxin Chen, Pavlo O. Dral

**Affiliations:** a State Key Laboratory of Physical Chemistry of Solid Surfaces, Department of Chemistry, College of Chemistry and Chemical Engineering, Fujian Provincial Key Laboratory of Theoretical and Computational Chemistry, Xiamen University Xiamen 361005 China dral@xmu.edu.cn; b Institute of Physics, Faculty of Physics, Astronomy, and Informatics, Nicolaus Copernicus University in Toruń ul. Grudziądzka 5 87-100 Toruń Poland; c Aitomistic Shenzhen 518000 China

## Abstract

Density functional theory (DFT) is the workhorse of reaction simulations, but it often suffers from either prohibitive cost or insufficient accuracy. In this work, we report AIQM2—the universal AI-enhanced QM method 2—as the first method that enables fast and accurate large-scale organic reaction simulations for practically relevant system sizes and time scales beyond what is possible with DFT. This breakthrough is based on the high speed of AIQM2, which is orders of magnitude faster than common DFT, while its accuracy in reaction energies, transition state optimizations, and barrier heights is at least at the level of DFT and often approaches the gold-standard coupled cluster accuracy. AIQM2 can be used out of the box without any further retraining. Compared to pure machine learning potentials, AIQM2 possesses high transferability and robustness in simulations without catastrophic breakdowns. We showcase the superiority of AIQM2 compared to traditional DFT by performing an extensive reaction dynamics study overnight and revising the mechanism and product distribution reported in the previous investigation of the bifurcating pericyclic reaction.

## Abstract 2

AIQM2's high speed, competitive accuracy, and robustness enable organic reaction simulations beyond what is possible with the popular DFT methods. It can be used for TS structure search and reactive dynamics, often with chemical accuracy.

## Graphical abstract

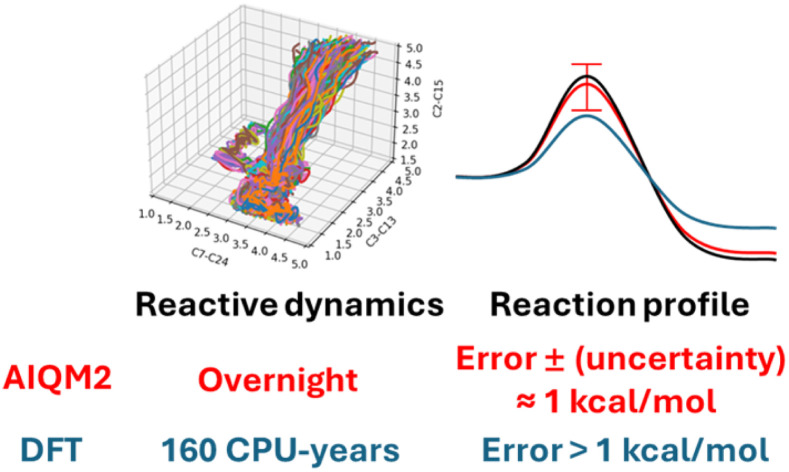

## Introduction

Reaction simulations provide atomistic-level information on the efficiency and mechanism of chemical transformations, guiding both experimental reaction design and *in silico* reaction planning. The common tools to simulate reactions are based on the concept of the potential energy surface (PES).^1^ Critical static points along the reaction pathway on the PES, such as minima (reactants, products, intermediates) and first-order saddle points (transition states, TS), can be used to derive kinetics and thermochemical properties. The key challenge is that to ensure high simulation accuracy, the PES ideally must be evaluated with chemical accuracy (1 kcal mol^−1^ error in energies), because the reaction rate depends exponentially on the Gibbs energy of activation according to the Eyring equation. Similarly, the product distribution under thermodynamic control strongly depends on the difference in the products' Gibbs free energies.

Minor changes to the shape of the PES, including the locations of critical points, can have large effects on reaction mechanisms and pathways,^2^*e.g.*, synchronous and step-wise mechanisms in the widely explored Diels–Alder reactions^3^ and ambimodal TSs in bifurcating reactions.^4^ To gain deeper insight into the conformational changes and energy flow during chemical transformations, the reaction dynamics must be investigated, where bonds breaking and formation are directly simulated by performing molecular dynamics (MD) trajectory propagations.^5^ One example is post-TS MD, which provides branching ratios and energy partitioning for reactions with diverging product channels—cases in which traditional Eyring's TS theory fails.^6^ While performing MD calculations is highly desirable for modeling reactions, an ensemble of reactive trajectories is usually required to obtain statistically robust and converged results, thus requiring computationally efficient methods for obtaining PESs.

**Fig. 1:**
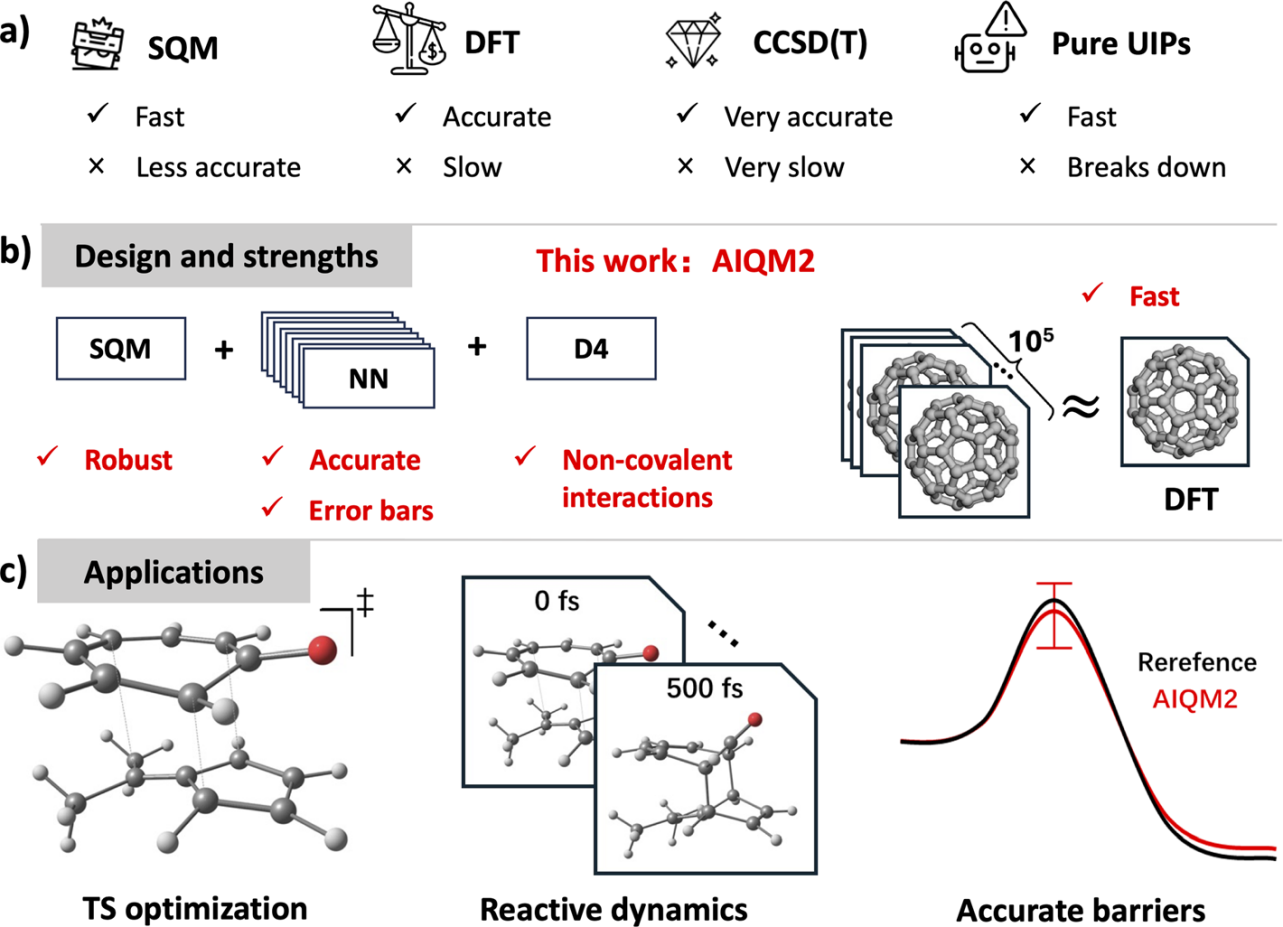
Selected types of methods used for simulating organic reactions. (a) Traditional QM methods exemplified by semi-empirical QM, density functional theory (DFT), the gold-standard CCSD(T), and universal (machine learning) interatomic potentials (UIPs)—each with its strengths and weaknesses. (b) AIQM2 proposed in this work eliminates the above weaknesses through careful design. (c) Various applications of AIQM2 in reaction simulations such as transition state (TS) optimization, reactive dynamics, and the accurate and fast calculation of reaction barriers.

Thus, the fast generation of well-shaped PES in the reactive region is required, which is a tall order for traditional quantum mechanical (QM) methods (Fig. 1a), because, generally, less expensive calculations are less accurate. For example, methods that achieve chemical accuracy on barrier heights—e.g., coupled-cluster methods and beyond,^7^ and double hybrid functionals with large basis sets^8^—may not be feasible for large systems and TS structure optimizations. The only practical solution widely adopted by the community is the combination schemes. In these schemes, cheaper and less accurate QM methods, such as commonly employed density functional theory (DFT) with relatively small basis sets or even semi-empirical QM methods, are used to obtain the geometries and frequencies of TS and minima. More expensive methods, such as the gold-standard CCSD(T),^9^ are then used to calculate the single-point energies on these geometries. These combination schemes are, obviously, approximations assuming that the geometry does not change much between the levels of theory, which is not necessarily true.^10^

The problem of high computational cost is particularly pertinent in reaction dynamics simulations, where one often must sacrifice the number of trajectories and, hence, the quality of the statistical analysis. Indeed, most reported studies propagated too few trajectories, leading to statistically unreliable product ratios,^11^ although, in these studies, accuracy has already been sacrificed by using relatively cheap DFT methods.

The rise of machine learning (ML) promises to speed up reaction simulations by directly predicting key properties, such as reaction outcome,^12^ yields,^13–16^ barrier heights,^17–27^ rate constants,^28,29^ as well as TS geometry.^30–37^ However, ideally, we would like to use the ML models as surrogate models to describe PES at the QM level. This would allow us to obtain similar levels of insight and to use existing, mature techniques for reaction simulations and analysis. Such models are popular ML interatomic potentials (MLIPs), where either an accurate description of the system-specific PES is established or universal interatomic potentials (UIPs) are constructed in the hope of covering the target chemical space.^38^

The construction of the system-specific MLIPs requires considerable effort because it requires careful selection of the training points on the PES and resource-intensive calculations with high-level reference QM methods.^39^ After all this work, these potentials cannot easily be reused for similar systems, and additional effort is needed to extend their applicability. A highly desirable alternative is UIPs, which promise to remove the need for repeated retraining while maintaining adequate accuracy for molecules outside the training set.

In principle, UIPs are pre-trained MLIP models, but constructing UIPs is much more challenging than training a system-specific MLIP because it requires an ML model with good transferability capabilities, large and representative datasets covering the chemical space of interest, as well as training and validation strategies ensuring good generalizing ability of the model, *etc.* Another important consideration in UIPs is the quality of the reference data. Most state-of-the-art (SOTA) UIPs target the DFT level because most of the available data are at the DFT level with relatively small basis sets.^40–46^ Hence, such potentials are bound not just to fail to achieve chemical accuracy in most cases but also to be inferior even to the reference DFT level they were trained on.

Only a few UIPs have gone beyond the DFT level and targeted the gold-standard coupled cluster level: AIQM1 (ref. 47) and ANI-1ccx^48^ are the first and only successful examples. AIQM1 is generally more transferable and robust than ANI-1ccx because AIQM1 is based on a Δ-learning^49^ approach, where MLIP is used only to correct the difference between the target (coupled cluster) and baseline (semi-empirical) QM levels, while ANI-1ccx is a pure MLIP model directly trained on the target coupled-cluster level. One of the major disadvantages of AIQM1 is its limitation to CHNO elements, which can be mitigated by using an extrapolative ONIOM approach,^50^ where AIQM1 is used for the major core region of the atomistic system, while lower-level methods such as ANI-2x^51^ and/or GFN2-xTB^52^ are used for the remaining parts containing other elements.

Both AIQM1 and ANI-1ccx generally achieve chemical accuracy within the scope of the training data, but we found that they have subpar performance in describing reaction barriers.^53^ Hence, until now, no theoretical method has been reported that can be used for organic reaction simulations—including transition state search, barrier height calculations, and reactive dynamics—with the robustness and transferability of DFT approaches, while being orders of magnitude faster.

Here, we introduce AIQM2, the second-generation general-purpose AI-enhanced quantum mechanical method, which approaches gold-standard coupled-cluster level accuracy at the cost of semi-empirical methods—i.e., beyond those of typical DFT approaches (Fig. 1b). As we show in this work, AIQM2 consistently improves upon its predecessor AIQM1 and the related universal ANI-1ccx method, especially for transition state optimizations and barrier heights. It also outperforms hybrid and double-hybrid DFT methods with a common double-ζ-quality basis set in reaction energies of large systems and some non-covalent interactions. In contrast to DFT, AIQM2 provides uncertainty estimates for its predictions, increasing the trustworthiness of the simulations with this method (Fig. 1b and c).

To showcase the efficiency and robustness of AIQM2, we propagated thousands of high-quality trajectories for a bifurcating pericyclic reaction in parallel on 16 CPUs within one day and obtained the product distribution at nearly coupled-cluster level accuracy. This way we effectively revise the original, much more expensive and less accurate, DFT results. AIQM2 is publicly available in our open-source software MLatom^54^ at https://github.com/dralgroup/mlatom.

To facilitate progress in ML-assisted computational chemistry, we have included the methods introduced in this work into the library of Universal and Updatable AI-enhanced QM foundational models (UAIQM).^55^ It is an umbrella platform that collects various models in a coherent library, enabling the automatic choice of the model according to the user's needs and, if required, the improvement of the models. UAIQM hosts both published (*e.g.*, AIQM1 and ANI-1ccx) and unpublished (*e.g.*, AIQM2, when this work was submitted) models so that the latest developments can be made accessible to the public timely.

## Results and discussion

### AIQM2 design

AIQM2 follows the same principle of Δ-learning^49^ as in AIQM1 by applying neural network corrections to the baseline semi-empirical QM method for higher accuracy at a lower cost. We have chosen GFN2-xTB^52^ as the baseline of AIQM2 due to its robustness and broad applicability in reaction exploration.^56^ In this regard, the change of baseline makes AIQM1 and AIQM2 two distinct methods, both in terms of composition and application preference. An explicit dispersion correction is added, because neither semi-empirical QM nor local neural network models can properly describe the long-range noncovalent interactions on their own.

Specifically, AIQM2 consists of 3 parts (Fig. 1b): the semi-empirical baseline GFN2-xTB* (asterisk indicates the special version of GFN2-xTB with D4 dispersion corrections^57^ removed), the correction predicted by the ensemble of 8 ANI neural networks,^58^ and the D4 dispersion correction (for the ωB97X^59^ functional). Thus, the energy predicted by AIQM2 can be formulated as:

1 *E*_AIQM2_ = *E*_GFN2-xTB^*^_ + *E*_NN_ + *E*_D4(ωB97X)_,

and energy derivative properties are the sum of each term's derivatives. Note that the Hessians provided by GFN2-xTB* and D4 are calculated by numerical differentiation on analytical gradients, while neural networks provide analytical Hessians; the final Hessian is the sum of these three Hessians. Other electronic structure properties, such as partial charges and dipole moments, are inherited from the baseline GFN2-xTB* calculations.

Several modifications were made to the original architecture used in ANI-1x and ANI-1ccx. The activation function was changed from CELU (Continuously Differentiable Exponential Linear Units) to GELU (Gaussian Error Linear Unit), which has been shown to provide better performance for higher-order energy derivatives.^60^ The angular cutoff in the atomic environment vector (AEV) was increased from 3.5 Å to 4.0 Å for a better description of long-range interactions during training and inference.^47^

In terms of the training data, the ANI model corrections were trained on the data derived from the ANI-1ccx and ANI-1x datasets.^61^ They were generated using an extrapolated coupled cluster method termed CCSD(T)*/CBS^61^ and a popular DFT method ωB97X^59^/def2-TZVPP,^62^ respectively. The asterisk in CCSD(T)* refers to the estimated TightPNO accuracy of DLPNO-CCSD(T)^63^ and the complete basis set extrapolation uses the formula by Halkier^64^ and Helgaker.^65^ Details about CCSD(T)*/CBS can be found in ref. 61. GFN2-xTB* data was generated for all the data points in both datasets and subtracted from the reference level to get the Δ-values^49^ for training. The models were first trained on the differences in energies and forces between ωB97X^59^/def2-TZVPP^62^ and GFN2-xTB*. This led to the AIQM2@DFT* approach approximating the ωB97X/def2-TZVPP level. To approximate the ωB97X-D4/def2-TZVPP level with explicit dispersion corrections, we can add the D4 corrections for the ωB97X functional, yielding the AIQM2@DFT method. The final models in AIQM2 were obtained by transfer learning on the energy difference between CCSD(T)*/CBS and GFN2-xTB*.^61^ The D4 corrections for the ωB97X functional were removed from the reference CCSD(T)*/CBS. Removing the D4 corrections during training was necessary because we added the explicit D4 corrections for the ωB97X functional back to the model when making predictions. In transfer learning, the first and the third layers were fixed as in ANI-1ccx and AIQM1, which effectively reduced the number of parameters for training and prevented overfitting to a smaller high-level dataset.

An advantage of AIQM2 is also that it provides uncertainty estimates of its predictions, in contrast to DFT approaches. The uncertainty of the calculation is defined as the standard deviation of the ANI models in the ensemble as is done for both AIQM1 and ANI-1ccx.^66^ We can interpret uncertainty as how different a new system, for which we want to make predictions, is from the molecules in the training set of AIQM2. The uncertainties are reported in this work as the error bars of the predictions, *i.e.*, energy estimates from averaging ANI model predictions ± standard deviation of the ANI models. The uncertainty for reaction energies is calculated as the standard deviation of the relative energies predicted by the ANI models. We calibrated the uncertainty on the standard CHNO dataset as done in the literature^66^ which yielded the threshold of 0.36 kcal mol^−1^, *i.e.*, the prediction is deemed reliable if the uncertainty is less than 0.36 kcal mol^−1^. From our previous tests on AIQM1 and ANI-1ccx, we have numerical evidence that highly uncertain predictions overall lead to bigger errors.^66^

### Revision of the bifurcating pericyclic reaction

**Fig. 2:**
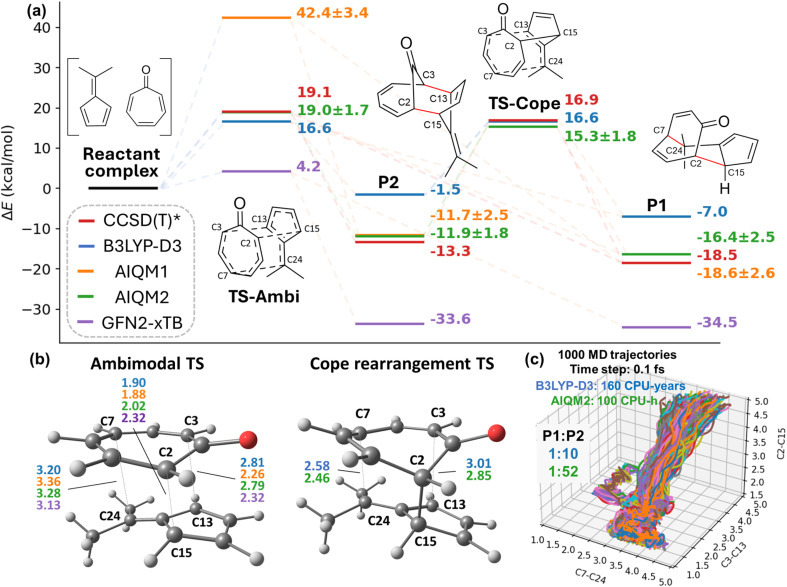
Energy profile, transition state geometries, and quasi-classical trajectories of ambimodal reaction. (a) Comparison of barrier heights and reaction energies in kcal mol^−1^ obtained with different methods. Energies are reported on the geometries optimized with the corresponding methods, except for CCSD(T)*/CBS energies evaluated on the B3LYP-D3/6-31G*-optimized geometries. Values after ± are the uncertainties of the relative energies given by AIQM2. (b) Critical bond lengths in angstroms (Å) of transition states optimized by various methods. The Cope rearrangement transition state is not found using GFN2-xTB and AIQM1, thus not presented. The same color scheme is used for each method as (a). (c) Distribution of 1000 quasi-classical trajectories starting from the ambimodal transition state (B3LYP product ratio is taken from ref. 67). B3LYP-D3 – B3LYP-D3/6-31G*, CCSD(T)* – CCSD(T)*/CBS, TS-Ambi – ambimodal TS, TS-Cope – Cope rearrangement TS.

Here we demonstrate the power of AIQM2 by revising the earlier state-of-the-art simulations^67^ of a paradigmatic pericyclic reaction, by performing resource-intensive downhill molecular dynamics (MD) simulations (Fig. 2). These simulations involve thousands of quasi-classical trajectories from the region around the ambimodal TS, leading to more than one product.

AIQM2 enables extensive explorations: overnight (100 CPU-hours) on commodity hardware, we could propagate 1000 quasi-classical trajectories, each 500 fs long in both forward and backward directions with 0.1 fs time step (in total, 10 million single-point evaluations, see computational details). These simulations on a smaller scale (only 117 reactive trajectories with 1 fs time step) were performed earlier^67^ with a much costlier DFT method B3LYP-D3/6-31G*, which would take 160 CPU-years for a thousand trajectories with 0.1 fs time step. Even with such extensive computational resources spent on DFT calculations, the number of trajectories might still have been insufficient to draw definite conclusions because of the low precision and potential for missing rare events, as shown in a recent analysis.^68^ AIQM2 simulations with 1000 trajectories, hence, furnish much higher statistical certainty.

**Fig. 3:**
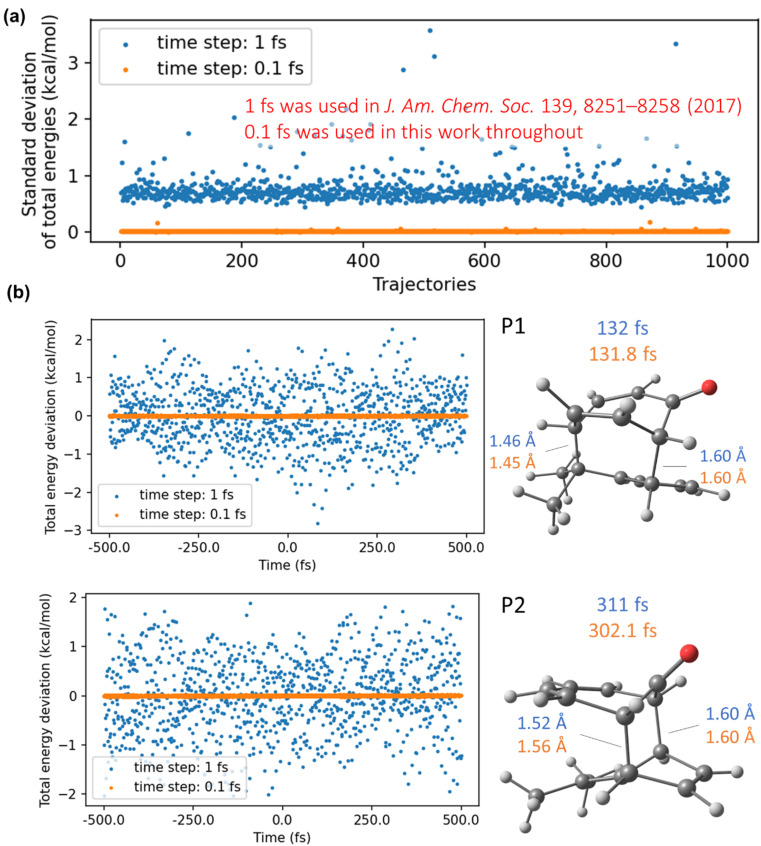
Total energies and their standard deviations along quasi-classical trajectories propagated with 1 fs and 0.1 fs using AIQM2. 1 fs was used in the original DFT study^67^ and is given here only for comparison; all results in this study are obtained with 0.1 fs time-step trajectories. (a) Comparison of the standard deviation of total energies in kcal mol^−1^ on 1000 trajectories propagated with 1 and 0.1 fs. (b) Comparison of total energy deviations with respect to the mean value on representative trajectories for P1 and P2. Figures on the right side show the snapshots where the products are first observed. Blue represents trajectories with the 1 fs time step, and orange represents those with the 0.1 fs time step. Time 0 fs corresponds to the initial geometry near the transition state.

In addition to propagating ten times more trajectories, we use the time step that is ten times smaller than in the original DFT study.^67^ Our AIQM2 simulations with 0.1 fs lead to energy-conservative dynamics, while we show that the 1 fs time step, employed in the original study, leads to rather large violations in energy conservation (Fig. 3b). The total energy fluctuations in 1 fs time-step trajectories reach up to several kcal mol^−1^, which might influence the conclusions drawn in the original DFT study. Indeed, our calculations with the same method, AIQM2, show that, *e.g.*, for an exemplary trajectory, product P2 is formed 9 fs later when the trajectory is propagated with 0.1 fs.

Most importantly, it is known^69^ that the quality of the downhill dynamics is strongly influenced by the accuracy of the QM method. Here our AIQM2 approach also has a significant advantage over B3LYP-D3/6-31G* as judged by the analysis of the PES stationary points. The barrier height of ambimodal TS at AIQM2 is almost the same as CCSD(T)*/CBS value. Note that the CCSD(T)*/CBS values are derived from DFT optimized geometries to avoid potential bias; we cannot compare to the geometries at the CCSD(T)* level due to its cost and absence of analytical energy derivatives. TS geometry optimized with AIQM2 qualitatively resembles that at B3LYP-D3/6-31G* with some noticeable differences in bond lengths. In comparison, B3LYP-D3/6-31G* underestimates the barrier by around 3 kcal mol^−1^. For the minima, the error between AIQM2 and reference CCSD(T)*/CBS is within *ca.* 2 kcal mol^−1^, while both B3LYP-D3 (*ca.* 10 kcal mol^−1^) and GFN2-xTB have huge errors (more than 25 kcal mol^−1^) compared to the same reference. Such large errors in B3LYP-D3 used in the previous study^67^ certainly influence the quality of the downhill dynamics.

Having at our disposal (1) a more accurate method, (2) more trajectories, and (3) better energy conservation, we turn to analyzing the simulation results. AIQM2 results differ from the DFT results in two major aspects. First, the product distribution changed from 1 : 10 to 1 : 52, showing that the formation of P1 has a much smaller role than previously thought. Second, the AIQM2 results show that the reaction proceeds *via* a dynamically stepwise mechanism rather than a concerted mechanism, as previously thought. The average time gap between the formation of the first and second bond is 205 fs for the major P2 product. The DFT study reported a 60 fs time gap. In AIQM2 trajectories leading to P2, the C3–C13 bond formation is delayed, and most of the C3–C13 bond lengths oscillate between 2.5 Å and 3 Å. This might potentially be caused by the PES curvature shaped by the “neighboring” TS corresponding to the Cope rearrangement connecting the competing products P2 and P1 (Fig. 2a and b). Once the first C2–C15 bond is formed, the formed adduct is hot and cannot immediately ‘decide’ which bond to form; eventually, the shorter C3–C13 bond is formed most of the time because it is typically shorter in the initial conditions. The role of the nearby TS is supported by the observation that the Cope rearrangement's barrier height at B3LYP-D3/6-31G* is much smaller than at AIQM2, which might explain the difference in product ratio. We did not observe the transformation of P2 to P1 in AIQM2 trajectories, as the barrier is rather high (27.24 kcal mol^−1^) compared to the small barrier at DFT (10.23 kcal mol^−1^).

Summarizing the analysis of the quasi-classical trajectories, we revised the previous state-of-the-art.^65^ While we leave more detailed explorations to future research, our dynamics study showcased the power of the AIQM2 approach, which should be considered instead of the common DFT methods when performing such simulations.

Several additional remarks are due. The B3LYP-D3/6-31G* calculations might have suffered from substantial basis-set superposition error (BSSE), which might have been rather large in TSs. An advantage of AIQM2 is that it was trained on the CCSD(T)*/CBS-level data, which were generated using a complete basis set (CBS) extrapolation scheme. By construction, CBS does not suffer from BSSE. AIQM2 can, therefore, provide a more accurate description in cases where BSSE becomes an issue for common approaches with the medium-sized basis sets like in B3LYP-D3/6-31G*.

Obviously, among the zoo of DFT functionals, there is always a high chance of finding a better functional than B3LYP/6-31G*. Particularly, M06-2X is now commonly used in the pericyclic reaction simulations because benchmarks showed its better accuracy for such simulations. For this particular reaction, M06-2X/6-31G* is more accurate than B3LYP-D3/6-31G* according to our comparison with CCSD(T)*/CBS (Table 1) and achieves similar accuracy to AIQM2. It does not help that M06-2X is even slower than B3LYP-D3, which would strongly hamper the extensive evaluation of many trajectories with small time steps. AIQM2 also provides error bars, allowing users to at least get an idea of how far the predictions might be from the target CCSD(T)/CBS values, while none of the DFT approaches offer such a handy feature.

**Table 1:** Comparison of various methods on energy profiles of ambimodal reaction and the estimated CPU time required to propagate 1000 trajectories with the 0.1 fs time step

	CCSD(T)*/CBS//B3LYP-D3/6-31G*	AIQM2//AIQM2	B3LYP-D3/6-31G*//B3LYP-D3/6-31G*	M06-2X/6-31G*//B3LYP-D3/6-31G*	M06-2X/6-31G*//M06-2X/6-31G*
Barrier (TS-Ambi)	19.1	19.0 ± 1.7	16.6	17.8	17.6
Reaction energy (P1)	−18.5	−16.4 ± 2.5	−7.0	−20.3	−19.7
Barrier (TS-Cope)	16.9	15.3 ± 1.8	16.6	15.5	15.4
Reaction energy (P2)	−13.3	−11.9 ± 1.8	−1.5	−14.2	−13.6
Time cost estimate for 1000 trajectories	—	100 CPU-hours	160 CPU-years	—	200 CPU-years

Finally, our approach has another huge advantage over common composite schemes, such as when semi-empirical methods are used for pre-screening possible transition states and calculating their ensembles, followed by refinement with the DFT methods. In our example of the pericyclic reaction, AIQM2 could find both the ambimodal and Cope rearrangement TSs, while the popular semi-empirical GFN2-xTB failed to locate the latter. In addition, GFN2-xTB has huge errors (more than 25 kcal mol^−1^) for the minima too. It means that the composite schemes relying on the semi-empirical methods are likely missing key conformers and TSs. Our approach is a big step forward in replacing the need for composite approaches by providing an all-in-one solution for all steps of the workflow from geometry optimization and ensemble sampling, to dynamics and ultimately reliable energetics.

### Overall AIQM2 performance

It is imperative to judge the quality of universal models by evaluating their performance on independent tests that are closer to the real-world applications. We impose a strict requirement on our new method to be applicable for performing molecular simulations with higher speed and with accuracy at least comparable to, or better than, DFT. Hence, we evaluate our new method on the GMTKN55 benchmark^8^ – a standard test for DFT methods. This benchmark covers various sizes of molecules and types of relative energies, which provides a comprehensive evaluation of AIQM2 beyond reaction properties. The reference values in the GMTKN55 benchmark are taken from high-level QM calculations which are expected to have errors within 1 kcal mol^−1^ relative to experiment. Hence, our evaluation will show the errors that might be expected when compared to the experiment.

**Fig. 4:**
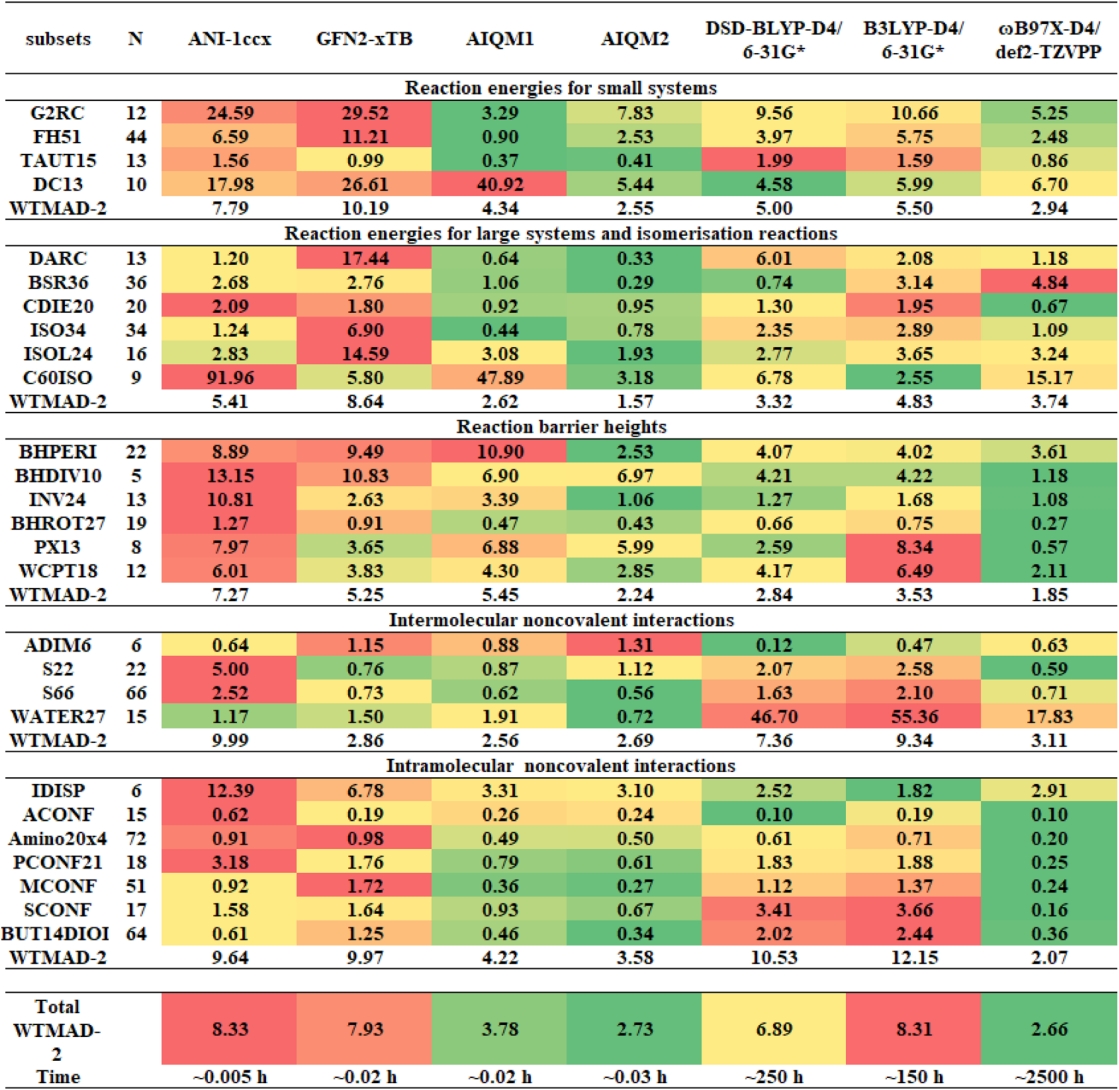
Performance assessment of AIQM2 compared to universal ML models ANI-1ccx and AIQM1 targeting coupled cluster accuracy and a selection of QM methods on the GMTKN55 data. Results are shown for neutral, closed-shell CHNO-containing compounds, as AIQM2 was designed to target coupled cluster accuracy on such types of molecules. Numbers are mean absolute errors in kcal mol^−1^, color-coded for each subset so that green corresponds to the smallest and red – to the largest errors (green – the best). WTMAD-2 is the weighted mean absolute deviation-2 in kcal mol^−1^ as defined in ref. 8. Time is reported in CPU hours. Notations and definitions of the reaction and interaction energies follow the original GMTKN55 benchmark paper;^8^ note that our benchmarks for DFT methods differ from the original ones, because we performed them with commonly used, smaller basis sets.

We compare the performance of AIQM2 to that of the DFT methods commonly used in practice, *i.e.*, B3LYP-D4/6-31G* and ωB97X-D4/def2-TZVPP; we also include a double-hybrid functional representative DSD-BLYP-D4/6-31G*. As is shown in Fig. 4, AIQM2 gives overall much better results than B3LYP-D4/6-31G* and DSD-BLYP-D4/6-31G* in terms of WTMAD-2, which is a weighted metric used to balance the different scales of various reaction energies. Note that the double-hybrid functional DSD-BLYP was recommended in the original GMTKN55 benchmark based on the evaluation with a much more expensive def2-QZVP, which is rarely used in practice. We instead choose the 6-31G* basis set for this functional from a practical perspective as one of the most commonly used in DFT studies, which is not intended for rigorous evaluation of the functional itself. Compared with the hybrid functional ωB97X with the more expensive triple-ζ basis set, AIQM2 can achieve comparable accuracy while maintaining the cost of a semi-empirical method. Overall, AIQM2 offers an outstanding cost-accuracy trade-off for predicting thermochemical, kinetic, and noncovalent properties: it is significantly faster than DFT approaches while maintaining competitive accuracy.

Considering the performance in each category, AIQM2 exhibits the lowest WTMAD-2 value of 1.57 kcal mol^−1^ among all tested methods for isomerization reaction energies of large systems. This is partly due to the good extensibility of the ANI neural network^70^ and the rich conformers presented in the training data, as exemplified in the performance of ANI-1ccx on the ISOL24 dataset. However, in cases where strong effects come from atoms outside the cutoff, such as the conjugated system, the ANI neural network may not work properly. Thus, baseline GFN2-xTB plays another important role in the robustness and accuracy of AIQM2. For example, in the C60ISO dataset, GFN2-xTB provides a good starting point for AIQM2 to approach mean absolute error (MAE) as low as 3.18 kcal mol^−1^, considering the average reaction energies in C60ISO are *ca.* 100 kcal mol^−1^. In the generally problematic non-covalent interactions, AIQM2 can even improve on some difficult cases for DFT, such as those in the WATER27 dataset, due to the extensive representation of water clusters in the training data, as known for the ANI-1ccx^48^ and AIQM1 (ref. 47) methods.

### Performance of AIQM2 for transition states and barrier heights

Another significant improvement of AIQM2 over related methods lies in its prediction of barrier heights. This presents a big challenge for AIQM1 and ANI-1ccx, as shown in the previous study,^53^ although they all target the CCSD(T)/CBS level. Compared to AIQM1, the WTMAD-2 of AIQM2 decreased from 5.45 to 2.24 kcal mol^−1^ for the datasets benchmarking the barrier heights (Fig. 4).

**Fig. 5:**
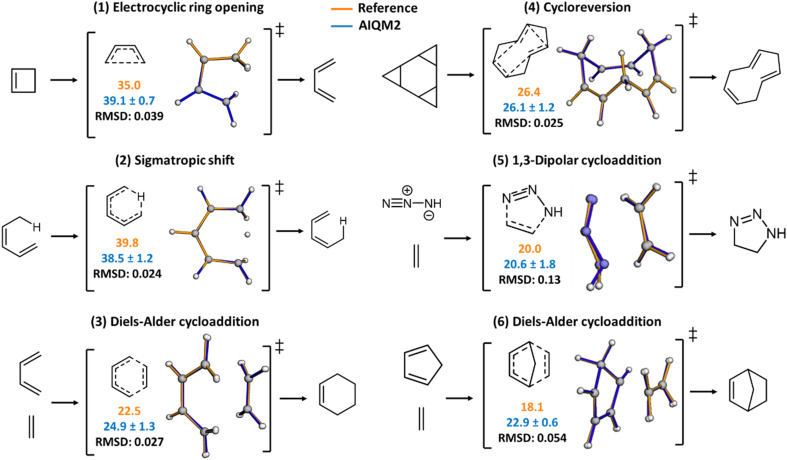
Representative reactions in BHPERI and their transition state structures. For the first three reactions, the reference barrier heights are approximated with the composite scheme to approach the CBS limit on the CCSD(T)/cc-pVTZ optimized geometries.^71^ The latter 3 reactions adopted reference values from the BHPERI dataset of the GMTKN55 2017 version, *i.e.*, barriers are calculated with W1–F12 and W2–F12 (ref. 72) methods on geometries from the CBS-QB3 (ref. 73) protocol. Orange is used for reference barrier heights, and blue for AIQM2. Barrier heights of AIQM2 are calculated using reference geometries to be consistent with the GMTKN55 benchmark. RMSD in Ångstrom shows the structural difference between the reference and the AIQM2-optimized TS. The same color scheme is used when overlapping their structures.

The most prominent change is in the BHPERI dataset, where the overall MAE is the lowest among the tested methods. Thus, we dive into the details of BHPERI and check whether AIQM2 can produce accurate geometries for TSs. Representative reactions in BHPERI and the comparison of barrier heights and TS geometries are shown in Fig. 5, which also demonstrates that the uncertainty of AIQM2 predictions is lower than that of the less accurate AIQM1.^53^ All TS structures were successfully found by AIQM2 and exhibited one imaginary frequency, with the normal mode vibration corresponding to the correct reaction.

Unfortunately, we cannot evaluate the quality of the TS geometry using the BHPERI dataset because it does not contain accurate reference geometries (which were optimized at B3LYP/6-31G* level). As a non-representative comparison, we report only the root-mean-square deviations (RMSDs) of three AIQM2-optimized TS structures relative to the CCSD(T)/cc-pVTZ geometries reported in a more recent work^71^ (Fig. 5): the Diels–Alder reaction starting from *s-cis*-butadiene and ethylene, the electrocyclic ring-opening reaction starting from cyclobutene, and the sigmatropic rearrangement originating from the *E*-isomer of 1,3-pentadiene. This recent work also reported higher-level energetics achieved with hierarchical focal point analyses (FPA), extrapolating to the *ab initio* limit. AIQM2 agrees well with the reference TS structures, with the RMSDs less than 0.1 Å.

## Conclusions and outlook

In this work, we report the second-generation general-purpose AI-enhanced Quantum Mechanical Method (AIQM2). AIQM2 offers remarkable speed and accuracy for reaction simulations by providing robust results for various applications out of the box. The overall accuracy of AIQM2 is rivaling the established DFT approaches, but the simulations are orders of magnitude faster. As we show, this can be exploited for efficient TS optimizations and extensive dynamics investigations of reaction mechanisms.

We exploited AIQM2's excellent performance to lift the accuracy in the quasi-classical downhill molecular dynamics, providing more statistically significant results with trajectories featuring energy conservation. This enabled us to obtain a new chemical insight into a bifurcating pericyclic reaction previously studied with fewer, non-energy-conserving trajectories obtained with a much slower and less accurate DFT approach. We show that this reaction proceeds *via* a stepwise mechanism rather than a concerted one, as previously believed. We also revise the product distribution in this ambimodal reaction to show that the minor product plays a much smaller role, which we attribute to the proximity of another TS corresponding to the Cope rearrangement, not described properly by B3LYP.

AIQM2 has an overall improved accuracy compared to its predecessor AIQM1, particularly for TSs and barrier heights, which are important for the quantum chemical reaction simulations. AIQM2 already has a growing track record of successful applications in real-world simulations beyond organic reactions, due to its universality, high speed, and accuracy. This method has been shown to provide outstanding IR spectra for a broad range of chemical compounds.^74^ Recently, it has also been used in the investigation of large systems, where AIQM2 enabled fast and accurate optimizations (*e.g.*, within 90 minutes on 32 GPUs for a 714-atom noncovalent complex), leading to high-quality UV/vis spectra calculated on these optimized geometries.^75^

All the power of AIQM2 is publicly available in MLatom at https://github.com/dralgroup/mlatom under the permissive MIT license, which lifts any limitations on the reuse of the method. The method is also included in the Aitomic package^76^ and can be used to perform online calculations on the Aitomistic Hub at https://aitomistic.xyz and XACS platform at https://XACScloud.com, further enhancing its availability.

## Computational details

All calculations were performed with the MLatom program.^54^ The D4 dispersion corrections were calculated *via* MLatom's interface to the dftd4 program,^77,78^ the ANI contributions *via* the interface to TorchANI,^58^ and the GFN2-xTB(*) calculations were performed *via* the interface to the locally modified xtb program.^79^ AIQM1 calculations require the ODM2* contributions which were calculated with the MNDO program^80^ interfaced to MLatom. MLatom was used for the B3LYP (VWN5) and ωB97X calculations *via* the interface to PySCF^81–83^ and DSD-BLYP *via* the interface to Orca program.^84,85^ Additional CCSD(T)*/CBS calculations were performed following the methodology in ref. 61 using the open-source MLatom implementation,^54^ where the required energy components were calculated *via* the interface to the Orca program.^84,85^

In the simulations of the bifurcating pericyclic reaction, we followed the simulation setup and analysis protocol analogous to those in the original study,^67^*i.e.*, initial conditions for quasi-classical trajectories were sampled from the harmonic quantum Boltzmann distribution starting at the ambimodal transition state, as implemented^86^ in MLatom.^54^ The trajectories were started near the TS region and essentially led to “downhill” dynamics to either products or reactants; forward and backward trajectories were launched from the same initial conditions, differing only in the sign of the initial velocities. Each set of such forward and backward trajectories, starting with the same initial conditions, yields a combined trajectory, where the time steps in the backward trajectories are reversed. Reactive trajectories were defined as those combined trajectories that contain reactants and one of the products; nonreactive combined trajectories contain either only reactants or only one of the products.

## Author contributions

P. O. D.: conceptualization, method design, original draft, figures. Y. C.: method development and implementations, calculations, analysis, manuscript revision, figures.

## Conflicts of interest

The authors declare no competing interests.

## Data Availability

AIQM2 is publicly available in the MLatom at https://github.com/dralgroup/mlatom. Data with energies and optimized coordinates for the performed benchmarks and ambimodal reaction analysis, as well as datasets used for training AIQM2, are available at https://github.com/707-Moira/aiqm2.
